# Influence of Plant Community Composition on Biomass Production in Planted Grasslands

**DOI:** 10.1371/journal.pone.0125758

**Published:** 2015-05-27

**Authors:** Max A. Henschell, Christopher R. Webster, David J. Flaspohler, Chad R. Fortin

**Affiliations:** School of Forest Resources and Environmental Science, Michigan Technological University, Houghton, MI, United States of America; University of Saskatchewan, CANADA

## Abstract

United States energy policy mandates increased use of renewable fuels. Restoring grasslands could contribute to a portion of this requirement through biomass harvest for bioenergy use. We investigated which plant community characteristics are associated with differences in biomass yield from a range of realistic native prairie plantings (n = 11; i.e., conservation planting, restoration, and wildlife cover). Our primary goal was to understand whether patterns in plant community composition and the Floristic Quality Index (FQI) were related to productivity as evidenced by dormant season biomass yield. FQI is an objective measure of how closely a plant community represents that of a pre-European settlement community. Our research was conducted in planted fields of native tallgrass prairie species, and provided a gradient in floristic quality index, species richness, species diversity, and species evenness in south-central Wisconsin during 2008 and 2009. We used a network of 15 randomly located 1 m^2^ plots within each field to characterize the plant community and estimate biomass yield by clipping the plots at the end of each growing season. While plant community composition and diversity varied significantly by planting type, biomass yield did not vary significantly among planting types (ANOVA; P >0.05). Biomass yield was positively correlated with plant community evenness, richness, C4 grass cover, and floristic quality index, but negatively correlated with plant species diversity in our multi-season multiple linear mixed effects models. Concordantly, plots with biomass yield in the lowest quartile (biomass yield < 3500 kh/ha) had 8% lower plant community evenness and 9% lower FQI scores than those in the upper quartile (biomass yield > 5800 kh/ha). Our results suggest that promoting the establishment of fields with high species evenness and floristic quality may increase biomass yield, while simultaneously supporting biodiversity.

## Introduction

Growing worldwide energy demands and the contributions of fossil fuel combustion to climate change have led to an interest in alternative renewable energy sources. In the United States, approximately 40% of energy needs are derived from imported oil and less than 15% comes from biofuels [1]. Currently, most biofuel (i.e. first-generation biofuel) is produced from *Zea mays* L. (corn) and other high oil content crops [2,3]. Use of these crops has been criticized for competing with food production for land and offering low overall reductions in greenhouse gas emissions [2,4]. The Energy Independence and Security Act of 2007 [5] mandates that 36 billion gallons of biofuels be available for transportation fuel, nearly 60% of which must be non-corn based by 2022. Second-generation biofuels, such as biodiesel and lignocellulosic ethanol from plants have the potential to provide a large portion of the non-corn biofuel requirements [6,7].

Native grasslands are among the most severely degraded ecosystems in North America with less than 4% of pre-Euro-American tallgrass prairie remaining [8]. Much of this land has been converted to agriculture. Partial restoration of agricultural lands has been pursued by planting native grassland species through programs such as the Conservation Reserve Program (CRP). Some fast growing perennial native grassland species, such as switchgrass (*Panicum virgatum* L.), have been identified as having great potential for the production of biomass and conversion to ethanol [6]. Such efforts represent an opportunity to re-establish some of the habitat characteristics and ecosystem services lost when tallgrass prairie was converted [7]. Tallgrass prairie planting for bioenergy feedstock production may be best suited for highly erodible land, marginal or abandoned agricultural land, and former wetlands currently used for agriculture [9]. A focus on such lands for bioenergy development would minimize competition with more productive agricultural lands currently used for food production. Restoration of these grasslands could also create high quality grassland habitat for wildlife [10].

Although numerous studies have investigated the relationship between plant species richness and productivity [11–15], fewer studies have investigated the response of plant productivity to other community metrics [12,16]. While controlled experiments of grassland plantings have shown a positive relationship between biomass yield and plant species richness, other studies of potential bioenergy crops have found that above-ground productivity decreases with species richness [14,15]. Other indices that account for species identity and community structure, such as floristic quality, diversity, and evenness metrics, may provide additional information about how community structure affects biomass yield.

For a plant community, Floristic Quality Index (FQI) takes into account the identity of each species and the species’ fidelity to the Pre-European plant community of the site [17]. High site FQI scores suggest a relatively intact relict or restored plant community typical of the region, site conditions, and natural disturbance regime. Consequently, sites with high FQI scores should be composed of co-evolved species that are well matched to local resources and environmental conditions. Such co-evolved native plant species assemblages have been shown to exhibit complementarity in resource use [18], which could contribute to greater biomass yield through transgressive overyielding [12], an important consideration when comparing different planting options for bioenergy production. This index, therefore, may provide a link between species richness and plant species complementarity.

Additionally, metrics that describe the abundance of species relative to each other and evenness within a community, such as the Shannon-Weaver Diversity Index (H′) [19] and a measure of plant species evenness with little *a priori* correlation with species richness [20], one of the most commonly used variables biomass yield studies, may help explain whether certain community structures produce more biomass in planted grasslands. For example, in some cases exotic species may outcompete native species for resources, causing a decrease in overall community complementarity [21] as well as FQI, community diversity and evenness.

Our objective was to determine which characteristics of the plant community were associated with biomass yield among a realistic suite of native prairie plantings. Specifically, we (1) investigated whether patterns in plant community composition were related to planting type and abiotic factors; (2) tested whether the Floristic Quality Index (FQI), species diversity (H'), species richness (S), species evenness (E') and biomass yield differed among grassland planting types and between years; and (3) related FQI, S, H', and E' of grassland plantings to biomass yield. We hypothesized that biomass yield would be positively associated with FQI, but measures of plant community richness and diversity would be associated with decreased biomass yield, as shown in some previous studies [14,15].

## Methods

### Study area

Our study was conducted in the south-central Wisconsin counties of Dane, Rock, and Iowa during 2008 and 2009. We selected 11 grassland fields from United States Fish and Wildlife Service (USFWS) Waterfowl Production Areas (WPA), Wisconsin Department of Natural Resources (WIDNR) wildlife areas, Conservation Reserve Program (CRP) fields, and privately owned tallgrass prairie restorations. WPA and WIDNR fields are primarily established for wildlife habitat. CRP fields are privately owned lands administered by the USDA Farm Service Agency (FSA), while tallgrass prairie restorations were established by the land owner. Fields averaged 8.8 (± 1.6) ha in size and were primarily mesic grasslands, however heavy rainfall in 2008 caused flooding in less than one-half of one field. These planting types were used as categorical variables in subsequent analyses: WPA, WIDNR, CRP, and restoration.

We obtained written permission from each landowner to access their property and conduct our research. A permit was required to access and sample CRP fields and was obtained from the USDA FSA.

### Plant community metrics

We sampled vegetation in 15 randomly located 1 m^2^ plots per field four times over the course of the 2008 and 2009 growing seasons: mid- to late May, mid- to late June, mid- to late July and late August to early September. During each visit, plant species were identified and percent cover was estimated by species. We recorded percent cover in 9 classes (<1%, 1–2%, 2–5%, 5–10%, 10–25%, 25–50%, 50–75%, 75–95%, >95%) [22]. For each plot, we calculated Shannon-Weaver Diversity Index (H′) as follows: ${\text{H}}^{\text{'}}=\text{-}\sum_{\text{i}}{\text{p}}_{\text{i}}\times \text{ln}{\text{p}}_{\text{i}}$, where *p*_*i*_ is the midpoint of the maximum cover class of the *i* ^th^ species recorded over one growing season [19,23]. We used the total number of species recorded within each growing season to determine species richness (S). We calculated species evenness as follows: $\text{E'}=\text{1-}\left(\sum_{\text{h}}^{\text{K}}{\text{p}}_{\text{ih}}\text{-}{\text{p}}_{\text{jh}}\right)/\text{S}$, where *p*_*ih*_ and *p*_*jh*_ are the midpoint of the maximum cover class recorded over the growing season for species *i* and *j*, and *K* is the number of binary differences between species within a plot: K=S(S-1)/2 [24]. We chose this measure of evenness because it has little *a-priori* correlation with species richness, was not correlated with H´ (r = 0.25), and meets the essential criteria for an ecologically relevant evenness index [20,25].

We also calculated a plot-level floristic quality index (FQI) each year. FQI is a commonly used, semi-quantitative index of plant community conservation value and is based on the fidelity of the resident species to natural plant associations characteristic of the habitat and natural disturbance regime [26–29]. The index is derived from coefficients of conservatism (*C*) of individual species [17], which are essentially estimates of niche breadth assigned by expert panels [29]. *C* ranges from 0 to 10, where a score of 0 is assigned to generalist or ruderal species with little if any fidelity to any particular intact plant association. Species with very limited niche breaths (often species of conservation concern) are assigned a score of 10. Non-native exotic species are also commonly assigned a score of 0 [29,30]. FQI is calculated as follows: $\text{FQI}=\overset{-}{\text{C}}\times \sqrt{\text{S}}$, where $\overset{-}{\text{C}}$ is the mean coefficient of conservation of a plot and *S* is the number of species found in a plot [17].

### Plant community productivity

We cleared all vegetation and litter from 15 randomly chosen 1 m^2^ plots in each field in the spring of 2007 prior to green up. Some plots were lost to human disturbance throughout the course of the study, primarily by private landowner’s management practices in accordance with CRP contract requirements (e.g., late season mowing or herbicide treatment of noxious weeds). The number of plots used for our biomass yield estimates ranged from 12 to 15 per field in 2008 (mean = 15) and 6 to 15 in 2009 (mean = 14). We clipped vegetation by hand in mid-October, retaining a stubble height of approximately 5 cm and leaving all litter in place, from all usable sample plots in late fall of 2007, 2008, and 2009. We clipped all plots within a field on the same day. We chose to collect vegetation at this time to mimic the likely timing of a biomass harvest [31]. We recorded the wet weight of clipped vegetation from each plot. We reserved a 1 L subsample from each clipped plot which was combined into a field-wide sample. We dried each field-wide sample in a drying oven at 70^°^C for 48 hrs [32] and recorded the field-wide sample dry weight. Plot-level above-ground dormant season biomass (hereafter biomass yield) was calculated as: $\text{biomass yield}={\text{D}}_{\text{f}}/{\text{W}}_{\text{f}}\times \;{\text{W}}_{\text{p}}\times 10,000$ where *biomass yield* is the estimated plot-level dry biomass yield (kg/ha), *W*_*f*_ is the field-wide sample wet weight (kg), *D*_*f*_ is the field-wide sample dry weight (kg), and *W*_*p*_ is the plot-level wet weight (kg/m^2^).

### Statistical analysis

To address our first objective, whether patterns of plant community composition were related to planting type, we performed nonmetric multidimensional scaling ordination (NMS) of the between plot rank-dissimilarity matrix for the square root of the species' cover class midpoints to explore patterns of plant community structure in our planting types [33]. We used data from both 2008 and 2009 in the ordination. We also fit environmental variable vectors to the NMS to investigate the relationship between planting types and environmental variables. We normalized the environmental variables prior to fitting vectors using the Wisconsin double standardization, which first standardizes the species-level data by maxima, then standardizes plot-level data by total [34]. The environmental variable vector indicates the direction of the most rapid positive change of the variable through ordination space, while the length of the vector indicates the correlation of the environmental variable to the plant community. For example, if a vector crosses through a plant community in ordination space, it is expected that the plant community will be positively associated with the environmental variable represented by the vector. We plotted the vectors of only those environmental variables that showed a strong correlation (*r* ^*2*^ > 0.50) to the plant community data in our analysis. We used analysis of similarity (ANOSIM) to test for differences in plant communities among planting types [35]. We calculated a Bray-Curtis dissimilarity matrix from the cover class midpoints for each plant species recorded in the field, which was used in ANOSIM. We used 999 Monte Carlo permutations to calculate the ANOSIM *R* statistic, which is an indicator of plant community similarity, where zero indicates identical communities while 1 indicates communities that are completely different. We considered p-values < 0.05 to be significantly different plant communities. NMS was completed in the R statistical environment [36], and ANOSIM was completed using Primer 6 [37].

Our second objective was to determine whether biomass yield, FQI, H′, S, E′, or C4 grass cover differed among grassland planting types and between years. We used two-factor analysis of variance, with α = 0.05, to test whether each variable differed between planting type and/or year. If there were differences between planting types and/or years, we used Tukey's honestly significant difference (HSD) test to identify those differences, at α = 0.05 [38].

We developed a set of *a priori* mixed effects multiple-linear regression models to quantify the relationship between biomass yield and FQI, H′, S, E′, and maximum plot-level cover of C4 grasses (Table 1). We selected 21 models nested within the global model, *biomass yield = a + FQI×x*_*1*_ *+ H′×x*_*2*_ *+ S×x*_*3*_ *+ E′×x*_*4*_ *+ C4×x*_*5*_, including the null and global models (Table 1). We employed a mixed effects modeling framework to account for our nested sample design where plots were nested within a field and plots were sampled in two consecutive years [39]. We log-transformed the variables FQI and E′ prior to regression analysis to correct for issues of non-normality. We added 1 to FQI values prior to log transformation, since some plots had a FQI of zero. The random effect used in our analysis was “field” in the models for 2008 and 2009, while “field” and “year” were random effects in the combined (hereafter 2008 + 2009) models. We used model averaging to estimate a final model for each time period (2008, 2009, 2008 + 2009). We used Schwarz's Bayesian Information Criterion (BIC) weight to average each model parameter found within the *a priori* model set with ΔBIC < 4 [40,41]. We used this model averaging framework to account for uncertainty in the true relationship between the chosen model parameters and plot-level biomass yield.

**Table 1:** Model parameter estimates, BIC, ΔBIC, and BIC_*w*_ results from top 5 univariate and multiple linear regression mixed model selection analysis, as well as the null and global models, investigating the relationship of biomass yield to C4 grassland cover (C4), plant species Shannon-Weaver Diversity Index (H′), plant species evenness (E′), Floristic Quality Index (FQI), and plant species richness (S).

	C4	H′	log(E′)	log(FQI+1)	S	BIC	ΔBIC	BIC_*w*_
2008	**36.35**					**2841.5**	**0**	**0.534**
	**38.66**		**726.3**			**2844**	**2.51**	**0.152**
	36.25				-25.33	2845.8	4.29	0.063
	35.87	-161.6				2846	4.53	0.055
	35.85			73.11		2846.4	4.92	0.046
	34.1	-1435	1701	536	69.03	2851.7	10.23	0.003
2009	**33.21**	**-2706**	**2777**		**223.1**	**2446.2**	**0**	**0.774**
	**29.34**	**-2930**	**2923**	**490.8**	**205.7**	**2449.1**	**2.91**	**0.18**
	34.46	-1276	1702			2452.8	6.65	0.028
	28.59	-1739	2021	704.6		2453.8	7.64	0.017
	24.89	-867.8				2461.4	15.27	0
						2478.8	32.57	0
2008 + 2009	**34.1**	**-2122**	**2405**		**177.1**	**5294.4**	**0**	**0.505**
	**29.56**	**-2362**	**2520**	**552.9**	**152.1**	**5294.9**	**0.53**	**0.388**
	**28.34**	**-1386**	**1717**	**704.3**		**5297.9**	**3.54**	**0.086**
	34.41	-863.8	1404			5300.8	6.43	0.02
	28.32	-534.5				5311.2	16.84	0
	34.33		793.1			5314	19.59	0
	30.31					5314.2	19.79	0

Models in bold were used in model averaging analysis.

## Results

We recorded 155 herbaceous species and 22 woody species in 2008 and 157 herbaceous species and 25 woody species in 2009 (S1 Table). The species that occurred in the most plots in both 2008 and 2009 was *Taraxacum officinale* F.H. Wigg followed by *Solidago canadensis* L. The most frequently occurring woody shrubs were *Rubus* spp., while trees were dominated by *Prunus serotina* Ehrh. The most common native C4 grass was *Andropogon gerardii* Vitman.

**Fig 1:**
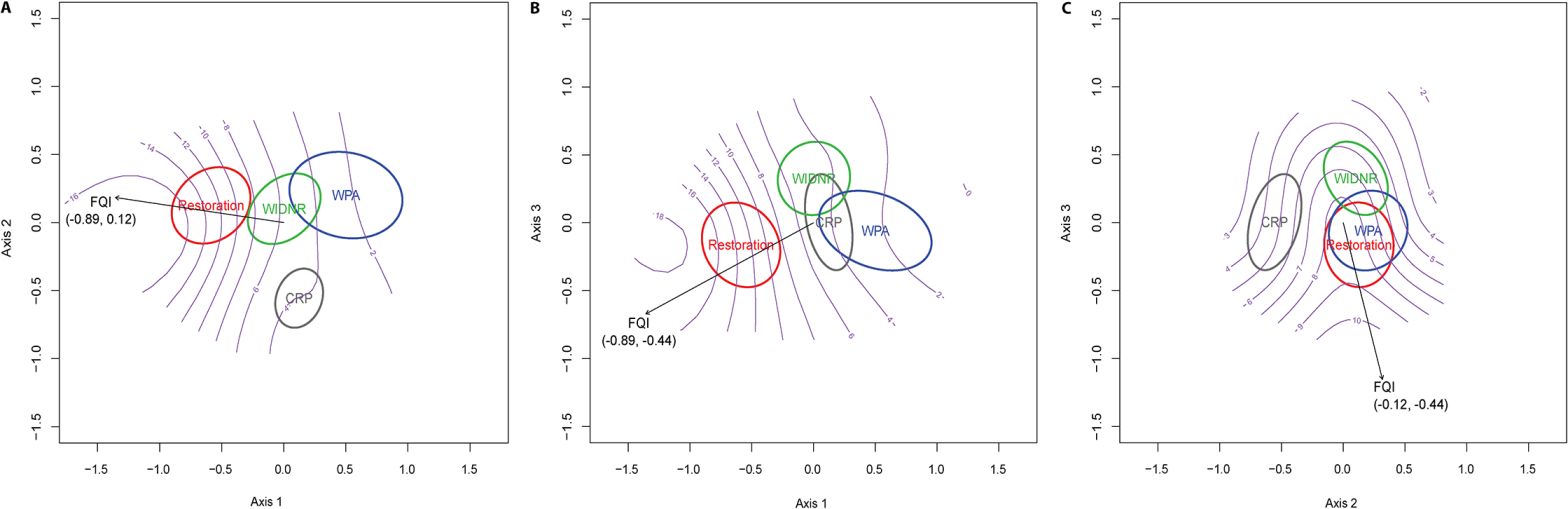
Nonmetric multi-dimensional scaling (NMS) biplots for vegetation in south-central Wisconsin prairies. A three axis solution best described the vegetation data: (A) axis 1 versus axis 2, (B) axis 1 versus axis 3, and (B) axis 2 versus axis 3. Ordination ellipses indicate planting type. Numbers under the environmental variable name represent the correlation with the horizontal and vertical axes of the respective plot. Purple gradients represent FQI scores.

The final 3-axis NMS solution had a stress value of 0.17. Restoration and CRP plots appeared to have the most distinctive plant communities in ordination space (Fig 1). ANOSIM results showed that all four planting types had distinctive plant community composition (Table 2). FQI was the only variable with r^2^ > 0.50 (Table 3). Restoration plots strongly associated with the high FQI values, while CRP plots were associated with intermediate FQI values (Fig 1).

**Table 2:** Analysis of similarities (ANOSIM) matrix for managed grassland plant communities among four planting types.

	Restoration	CRP	WPA	WIDNR
Restoration	—-	<0.001	<0.001	<0.001
CRP	0.73	—-	<0.001	<0.001
WPA	0.50	0.46	—-	<0.001
WIDNR	0.45	0.58	0.30	—-

Numbers below the diagonal are ANOSIM *R* values, those above are P-values. ANOSIM *R* values typically range from zero to one, where zero are completely dissimilar communities and one represents completely similar communities.

**Table 3:** Correlation of environmental variables with nonmetric multidimensional scaling axes.

Environmental Variable^†^	Axis 1	Axis 2	Axis 3	r^2^	P
Biomass yield	0.87	0.47	-0.13	0.33	< 0.001
S	-0.52	-0.80	0.30	0.30	< 0.001
H′	-0.66	-0.62	0.42	0.32	< 0.001
**FQI**	**-0.89**	**0.12**	**-0.44**	**0.72**	**< 0.001**
E′	0.92	0.38	0.10	0.45	< 0.001

Bold indicates environmental variables with r^2^ > 0.50, whose vectors are shown on nonmetric multidimensional scaling plots.

^†^Variable definitions: S: plant species richness; H′: plant species Shannon-Weaver diversity index; FQI: Floristic Quality Index; E′: plant species evenness.

**Fig 2:**
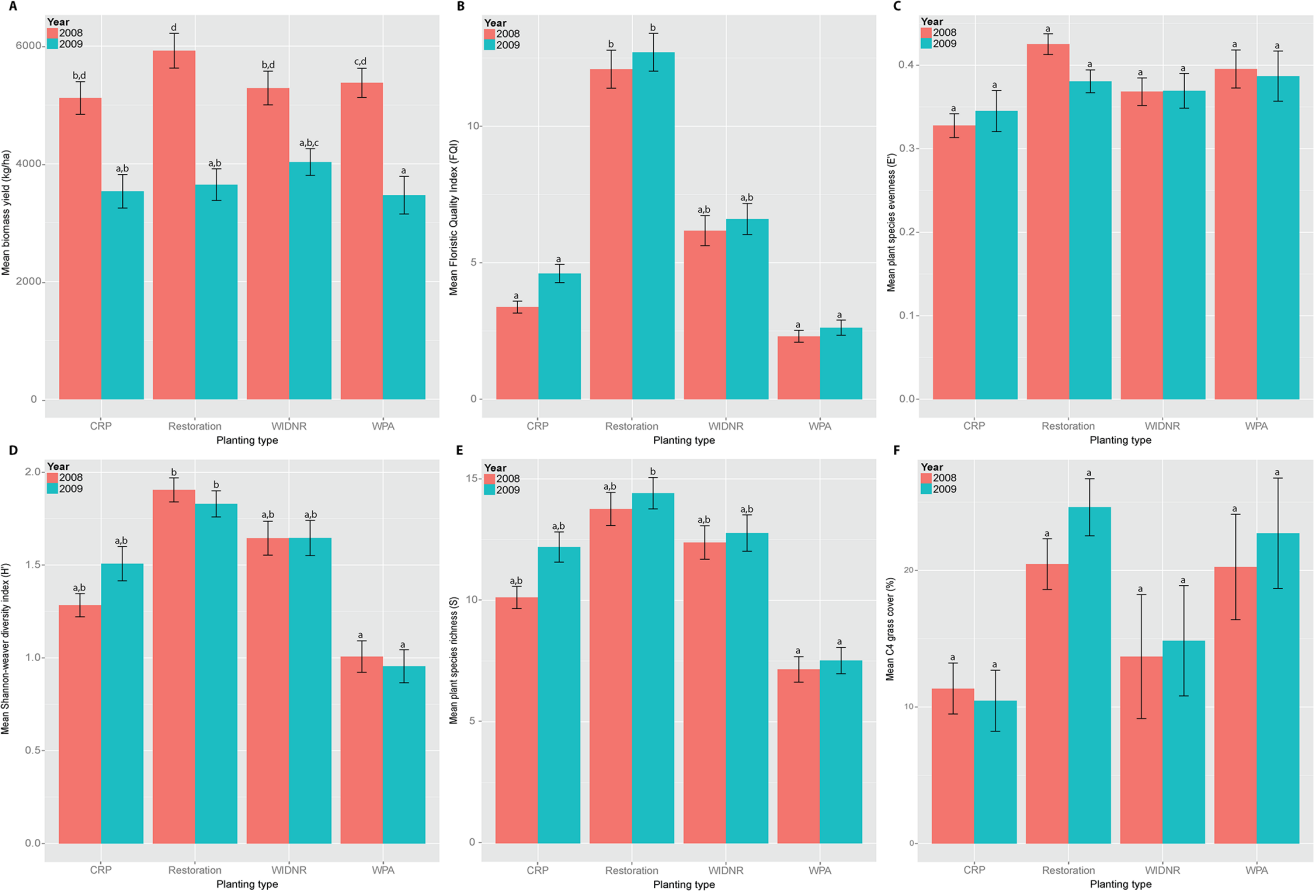
Box plots of vegetation characteristics by planting type and year. Letters indicate means that are significantly different (Tukey’s HSD, P < 0.05) for (A) biomass yield (kg/ha), (B) Floristic Quality Index (FQI), (C) Evenness (E′), (D) Shannon-Weaver Diversity Index (H′), (E) species richness (S), and (F) C4 grass cover among planting types and between years.

Plot-level biomass yield estimates ranged from 1455.6 kg/ha to 10,437.7 kg/ha in 2008 (mean = 5446 ± 141.00; Fig 2, S2 Table) and from 790.4 kg/ha to 10,706.9 kg/ha in 2009 (mean = 3655.8 ± 146.96; Fig 2, S2 Table). Biomass yield did not differ among planting types (*F(3*,*291)* = 0.68, *P* = 0.56; Fig 2), but did differ between years (*F(1*,*291)* = 78.90; *P* < 0.001; Fig 2). Plot-level FQI ranged from 0 to 21.5 in 2008 (mean = 6.0 ± 0.39; Fig 2, S2 Table), and 0 to 24.1 in 2009 (mean = 7.0 ± 0.44; Fig 2, S2 Table). FQI did differ by planting type (*F(3*, *291)* = 175.2, *P* < 0.001; Fig 2), but did not differ between years (*F(1*,*291)* = 2.82, *P* = 0.09; Fig 2). Plots with 0 FQI were typically dominated by exotic species. Plot H′ ranged from 0 to 2.8 in 2008 (mean = 1.5 ± 0.05; Fig 1, S2 Table) and from 0 to 2.7 in 2009 (mean = 1.5 ± 0.05; Fig 2, S2 Table). H′ did differ among planting types (*F(3*, *291)* = 51.3, *P* < 0.001; Fig 2), but did not differ between years (*F(1*,*291)* = -0.006, P = 0.94; Fig 2). One plot had zero-value H′ in 2009 and three plots had zero-value H′ in 2009. These plots consisted solely of the exotic *Phalaris arundinacea* L. Plot-level S ranged from 1 to 23 species in 2008 (mean = 10.7 ± 0.36; Fig 2, S2 Table) and from 1 to 29 species in 2009 (mean = 11.5 ± 0.40; Fig 2, S2 Table). S differed among planting types (*F(3*, *291)* = 50.24, *P* < 0.001; Fig 2), but did not differ between years (*F(1*,*291)* = 3.12, P = 0.08; Fig 2). Plot-level E′ ranged from 0.18 to 1.0 species in 2008 (mean = 0.38 ± 0.01; Fig 2, S2 Table) and from 0.17 to 1.0 species in 2009 (mean = 0.37 ± 0.01; Fig 2, S2 Table). E′ did differ among planting types (one-way ANOVA; *F(3*, *291)* = 4.31, *P* = 0.005; Fig 2), but did not differ between years (*F(1*,*291)* = 0.75, *P* = 0.39; Fig 2). C4 grass cover ranged from 0.00 to 86.5 in 2008 (mean = 16.78 ± 1.54; Fig 2, S2 Table) and from 0 to 86.5 in 2009 (mean = 19.77 ± 1.76; Fig 2, S2 Table).

Two models investigating the association of biomass yield to plant community characteristics had ΔBIC < 4 in 2008 and 2009, and three models in 2008 + 2009. C4 grass cover was in every model with ΔBIC < 4 in all time periods (Table 1). The best performing model in 2008 (ΔBIC = 0) contained only one variable, C4 grass cover, while the second best performing model (ΔBIC = 2.51) contained two variables, C4 grass cover and E′ (Table 1). C4 grass cover and E′ were both positively associated with biomass yield (Table 1). The best performing model in 2009 and 2008 + 2009 contained C4 grass cover, H′, E′, and S (Table 1). C4 grass cover, E′, and S were all positively associated with biomass yield, while H′ was negatively associated with biomass yield (Table 1). The second best performing model in 2009 and 2008 + 2009 (ΔBIC = 2.91 and ΔBIC = 0.53, respectively) was the full model. C4 grass cover, E′, FQI, and S were all positively associated with biomass yield in both 2009 and 2008 + 2009, while H′ was negatively associated with biomass yield in both time periods (Table 1).

**Fig 3:**
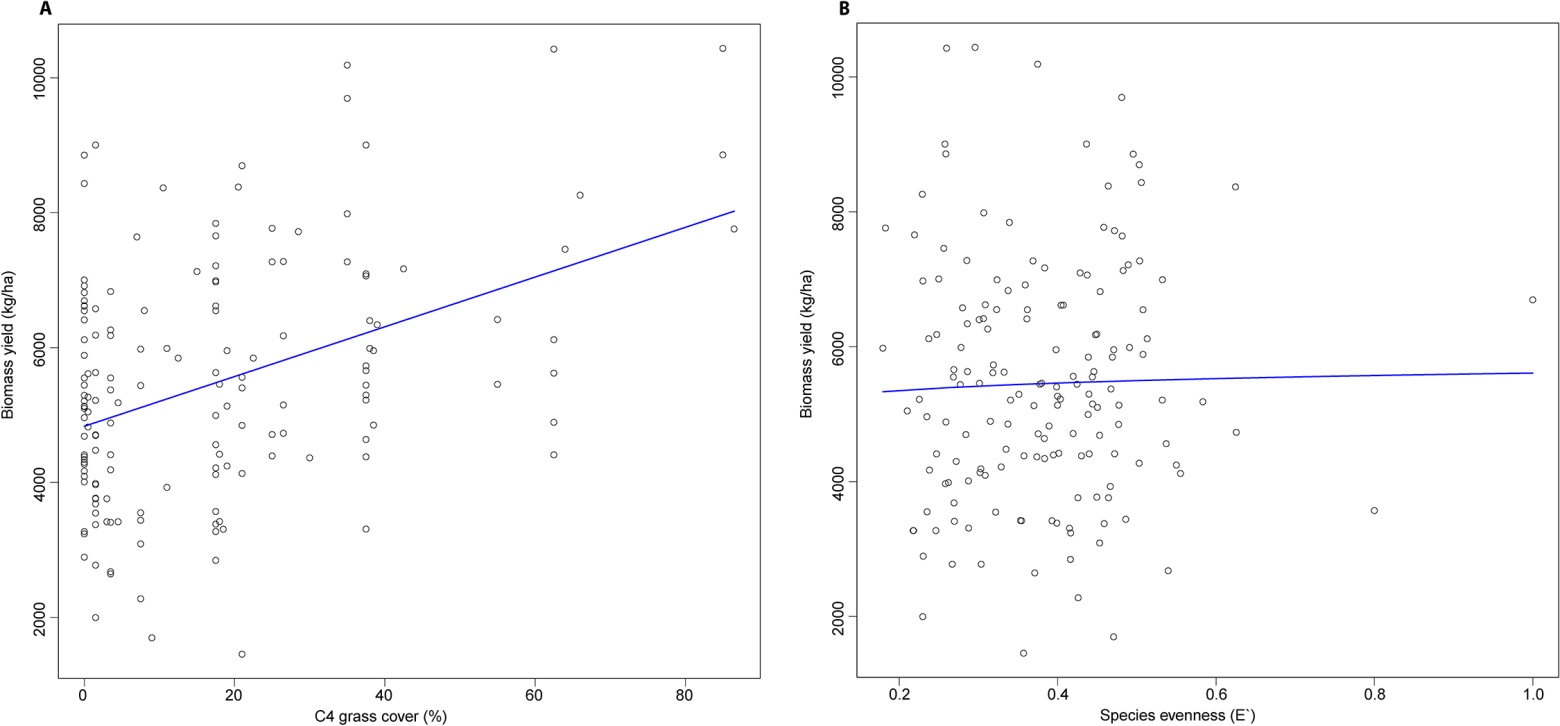
Variable plots for the model average for 2008 data. The regression line for multiple regression is displayed as the intercept and slope for the indicated predictor variable with the mean of all other variables held constant. Coefficients for each model are listed in Table 4. Log-transformed variables have been back-transformed. (A) The relationship between the percent of C4 grass cover within 1 m^2^ plots and biomass yield (kg/ha). (B) The relationship between species evenness within 1 m^2^ plots and biomass yield (kg/ha).

**Fig 4:**
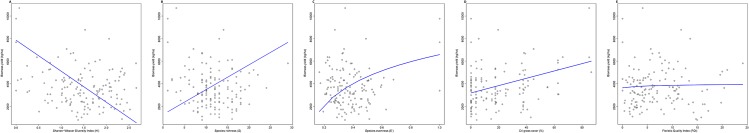
Variable plots for the model average for 2009 data. The regression line for multiple regression is displayed as the intercept and slope for the indicated predictor variable with the mean of all other variables held constant. Coefficients for each model are listed in Table 4. Log-transformed variables have been back-transformed. (A) The relationship between the Shannon-Weaver diversity index (H´) for the plant community within 1 m2 plot and biomass yield (kg/ha). (B) The relationship between plant species richness (S) within 1 m^2^ plots and biomass yield (kg/ha). (C) The relationship between plant species evenness (E´) within 1 m^2^ plots to biomass yield (kg/ha). (D) The relationship between the percent of C4 grass cover within 1 m^2^ plots to biomass yield (kg/ha). (E) The relationship between the Floristic Quality Index (FQI) within 1 m^2^ plots to biomass yield (kg/ha).

**Fig 5:**
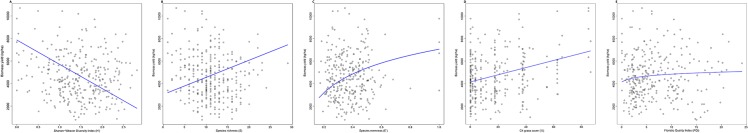
Variable plots for the model average for 2008 + 2009 data. The regression line for multiple regression is displayed as the intercept and slope for the indicated predictor variable with the mean of all other variables held constant. Coefficients for each model are listed in Table 4. Log-transformed variables have been back-transformed. (A) The relationship between the Shannon-Weaver diversity index (H´) for the plant community within 1 m2 plot and biomass yield (kg/ha). (B) The relationship between plant species richness (S) within 1 m^2^ plots and biomass yield (kg/ha). (C) The relationship between plant species evenness (E´) within 1 m^2^ plots to biomass yield (kg/ha). (D) The relationship between the percent of C4 grass cover within 1 m^2^ plots to biomass yield (kg/ha). (E) The relationship between the Floristic Quality Index (FQI) within 1 m^2^ plots to biomass yield (kg/ha).

Because of the relatively few models with ΔBIC < 4, our model averaging results closely followed the results from individual models. The model average for 2008 contained C4 grass cover and E′, both of which were positively associated with biomass yield (Table 4, Fig 3). All five predictor variables were found in the model average for 2009 and 2008 + 2009 (Table 4, Figs 4 and 5). C4 grass cover, FQI, S, and E' were positively associated with biomass yield, while H' was consistently negatively associated with biomass yield in the 2009 and 2008 + 2009 model average (Table 4, Figs 4 and 5).

**Table 4:** Parameter estimates, adjusted standard errors (SE), and z-values for model averaging analysis within each time period.

	Parameter^†^	Estimate	SE	Adj SE	z-value	Variable importance
2008	Intercept	4989.8	392.506	394.014	12.664	—-
	C4	36.864	6.775	6.829	5.398	0.69
	log(E)	726.272	455.946	459.731	1.58	0.15
2009	(Intercept)	7234.33	755.90	763.26	9.48	—-
	H	-2747.99	506.99	511.81	5.37	0.95
	S	219.78	65.87	66.51	3.31	0.95
	log(E)	2804.14	541.85	547.10	5.13	0.95
	C4	32.48	6.88	6.94	4.68	0.95
	log(FQI+1)	490.78	347.71	351.13	1.40	0.18
2008 + 2009	(Intercept)	7360.34	929.34	933.15	7.89	—-
	H	-2152.65	477.17	478.64	4.50	0.98
	S	166.25	52.16	52.38	3.17	0.89
	log(E)	2390.08	486.45	488.18	4.90	0.98
	C4	31.794	5.472	5.492	5.789	0.98
	log(FQI+1)	580.39	243.986	245.009	2.369	0.47

Two models were used in determining parameter values for 2008 and 2009 (Table 1), while three models were used in determining parameter values for 2008 + 2009 (Table 1).

^†^ **Parameter definitions: C4: C4 grass cover; S: plant species richness; H′: plant species Shannon-Weaver diversity index; FQI: Floristic Quality Index; E′: plant species evenness.**

## Discussion

Our findings suggest that biomass yield is related to a number of plant community metrics, such as species evenness, richness and floristic quality (FQI), which were all positively associated with biomass yield, and plant species diversity, which was negatively associated with biomass yield. Initial differences in seed mix composition likely had a persistent influence on community composition among planting types. Restoration and CRP plots were most similar in community composition. In both types, woody vegetation and exotic species were usually controlled, typically to preserve tallgrass prairie character in the former and in accordance with CRP land contracts in the latter. Restorations were also the most species-rich planting type, and are therefore most likely to have overlapping plant communities with other planting types. We found this to be true in our ANOSIM results, where similarity of restorations ranged from 0.45–0.73 with other planting types (Table 2). Prairie restorations are also planted with seed mixes from species with a high average *C*, since these species typify a native tallgrass prairie. It is not surprising then that restorations were associated with high FQI in the ordination.

The lack of differences in S, H′, FQI, and E′ between years was not surprising given that fields dominated by perennial species tend to change composition only slowly or following major physical disturbances [42,43]. Field initiation with a high diversity seed mix may reduce their susceptibility to invasion by invasive exotic species [44]. Since exotic species are scored a zero (*C* = 0) in our calculation of FQI, their removal or prevention increases FQI.

The upper limits of our productivity estimates were nearly three times higher than grassland biodiversity experiments in North America and were higher than similar experiments in northern Europe [11–13,45], but our estimates were similar to those of natural grasslands studied as potential sources of bioenergy feedstocks (S4 Table) [14,46–48]. These differences may be an artifact of scaling up biomass yield from 1 m^2^ plots to hectare-level yield. Weigelt et al. [43] found that the variability in biomass yield within a plot increased as the size of the plot decreased, and attributed this to small-scale differences within a field such as herbivorous insect and rodent activity as well as soil nutrient levels and structure. Di Virgilio et al. [49] found that the biomass yield of *P*. *virgiatum* varied over 8-fold within a field and attributed this to variation in soil fertility. Another possible discrepancy in biomass yield comes from the harvest technique: Monti et al [50] found differences in hand-harvested versus machine-harvested biomass yield, and attributed their findings to higher remaining stem height in mechanically harvested fields and biomass left in the field when mechanically collected. We also speculate that differences in biomass yield among plots may be influenced by spatial variability in the distribution of high yielding species. For example, high-yielding bunchgrasses, such *A*. *gerardii*. and *P*. *virgatum*, are often distributed irregularly. Consequently, by chance, some 1 m^2^ plots contained an entire bunch, while others contained only a portion or missed capturing a bunch altogether. This could result in scaling issues, particularly when compared to larger plots. Our finding that biomass yield did not differ across planting type suggests that the general practices used to manage these fields as grasslands has little effect on biomass yield.

C4 grass cover was positively associated with biomass yield in our study, which is likely due to their tall growth form (up to 3 m). Dominance by high-yield C4 grasses, such as *P*. *virgatum*., *A*. *gerardii*, and *Sorghastrum nutans* (L.) Nash, has been associated with increased biomass yield [14,42,51], increased ethanol yield from bioenergy crops [14], and increased ethanol production efficiency [52]. Consequently, maintaining a matrix of these species is an important component of native grassland bioenergy feedstock production.

FQI was also positively associated with greater biomass yield, particularly in our multi-season analysis. FQI is based on the identity of species present and their associated *C* scores, but does not include the abundance of individual species (i.e., percent cover) in the calculation. Consequently, the addition of a few species with high *C* scores may have a disproportionate effect on estimated FQI and have a similar FQI as a community that includes several species of intermediate *C*. Interestingly, both situations were associated with greater biomass yield than plots dominated by exotic or ruderal species. High biomass yield in plots with high *C* species, however, is likely associated with species complimentarity, rather than their productivity *per se*, and may result in transgressive overyielding [12].Cardinale et al. [53] also found that the inclusion of high *C* species can result in greater plot-level biomass yield than would be predicted based on their additive contribution to communities containing high-yielding C4 grasses. Most of the high *C* species in our system were low-biomass yield forbs. Consequently, low-biomass, high *C* species, which have co-evolved with high biomass yield species as a result of efficient resource partitioning [54], may be an overlooked component in the design of grasslands established for bioenergy feedstock production, and may suggest a mechanism for why native plant communities are more productive.

Retaining or planting native perennial bioenergy crops might result in decreased biomass yield within a field when compared to first-generation biomass crops such as *Z*. *mays*, but provides numerous other ecosystem services [15]. Native perennial bioenergy fields provide breeding and migratory stopover habitat for grassland birds [55,56], one of the most imperiled bird communities in the Unites States. Native perennial fields also provide habitat for predatory arthropods, important for pest suppression in other agricultural crops, and for pollinators of agricultural crops [15]. Interplanting strips of native perennials within conventional agricultural crops also results in improved soil and water conservation and nutrient retention within fields, which results in decreased inputs, and cost, into these conventional systems [57].

Promoting high field-level species evenness and FQI, in conjunction with maintaining a high cover of C4 grasses, may increase biomass yield, and provide benefits that extend beyond the field as well. Our results suggest that promoting species richness and floristic quality in grassland bioenergy plantings may enhance overall yields.

## Supporting Information

S1 Table Mean percent cover of plant species found in south-central Wisconsin grasslands. *C* is the coefficient of conservatism used to calculate FQI.
(XLSX)

S2 Table Summary of plot-level metrics used in analysis. (XLSX)

S3 Table Plot-level species percent cover used in nonmetric multidimensional scaling. Species codes are listed in S1 Table.
(XLSX)

S4 Table Biomass yield comparison of recent biodiversity-ecosystem function experiments and grassland studies. (XLSX)
